# The *Entamoeba histolytica* Syf1 Homolog Is Involved in the Splicing of AG-Dependent and AG-Independent Transcripts

**DOI:** 10.3389/fcimb.2018.00229

**Published:** 2018-07-09

**Authors:** Diana M. Torres-Cifuentes, José M. Galindo-Rosales, Odila Saucedo-Cárdenas, Jesús Valdés

**Affiliations:** ^1^RNA Laboratory, Department of Biochemistry, Centro de Investigación y de Estudios Avanzados del Instituto Politécnico Nacional, Mexico City, Mexico; ^2^Departamento de Histología, Facultad de Medicina, Universidad Autónoma de Nuevo León, Monterrey, Mexico; ^3^División de Genética, Centro de Investigación Biomédica del Noreste, Instituto Mexicano del Seguro Social, Monterrey, Mexico

**Keywords:** Xab2, Prp19C, splice sites, transcription, nuclear, CTD, human parasite

## Abstract

Syf1 is a tetratricopeptide repeat (TPR) protein implicated in transcription elongation, spliceosome conformation, mRNA nuclear-cytoplasmic export and transcription-coupled DNA repair. Recently, we identified the spliceosomal components of the human parasite *Entamoeba histolytica*, among them is EhSyf. Molecular predictions confirmed that EhSyf contains 15 type 1 TPR tandem α-antiparallel array motifs. Amoeba transformants carrying plasmids overexpressing HA-tagged or EhSyf silencing plasmids were established to monitor the impact of EhSyf on the splicing of several test Entamoeba transcripts. EhSyf Entamoeba transformants efficiently silenced or overexpressed the proteins in the nucleus. The overexpression or absence of EhSyf notably enhanced or blocked splicing of transcripts irrespective of the strength of their 3′ splice site. Finally, the absence of EhSyf negatively affected the transcription of an intron-less transcript. Altogether our data suggest that EhSyf is a bona fide Syf1 ortholog involved in transcription and splicing.

## Introduction

The spliceosome carries out co-transcriptional pre-mRNA (pre-messenger RNA) splicing (Wahl et al., 2009) with dynamical and sequential assembly of subunits on the pre-mRNA substrate for every splicing episode (Wahl and Lührmann, 2015) by, identifying conserved intronic elements. Whereas the U1 snRNP (small nuclear ribonucleoprotein) first binds to the 5′ss (splice site), SF1 (splicing factor 1) and U2AF65/35 (U2 snRNP auxiliary factors of 65 and 35 kDa) bind the BS (branch point sequence), the pY (polypyrimidine tract) and the 3′ss, respectively, thus forming complex A. Next, the small RNA molecule of U2 snRNP replaces SF1 forming a double stranded RNA hybrid with the BS, bulging the conserved A of the branch necessary for the first of the two transesterification reactions involved in the elimination of the intron-lariat and splicing of exons. Upon entry of the U4/U6.U5 tri-snRNP complex B is formed, which becomes an active spliceosome (B^ACT^) when U4/U6 basepairing is unwound allowing U6 snRNA to form two mutually exclusive interactions with the 5′ss and U2 snRNA, destabilizing U1 and U4 snRNPs. The first transesterification reaction occurs when U6/U2 and U2/BS RNA/RNA interactions position the bulged BS adenosine to attack the 5′ss, producing 5′ exon and intron lariat-3′ exon intermediates. The active spliceosome undergoes various rearrangements to form complex C that carries out the second transesterification reactions, ligating the exons and removing the intron-lariat (Wahl et al., 2009).

Other non-snRNPs factors are major players in the splicing process (Wahl et al., 2009), among them the components of the Prp19 complex or NTC in yeast: Prp19, Cef1, Syf1, Syf2, Syf3, Snt309, Isy1, and Ntc20 (Fabrizio et al., 2009). Prp19 is the scaffold for NTC formation, and it is essential for splicing but does not constitute any of the individual spliceosomal snRNPs (Tarn et al., 1993a,b, 1994). The NTC joins the spliceosome before, or during, U4/U6 unwinding and remain associated with the spliceosome during the two steps of splicing, marking the change from the inactive B to the active B^ACT^ spliceosome (Chan et al., 2003). Recent evidence shows that NTC regulates spliceosome conformations and fidelity (Hogg et al., 2010), and as part of the intron lariat complex participates in spliceosome disassembly (Fourmann et al., 2013).

It has been shown that U2AF65 and NTC interact both *in vitro* and *in vivo*, and this interaction is required for activation of splicing (David et al., 2011). U2AF65 binds directly to the Ser2 phosphorylated carboxy terminus domain of RNA Pol II (CTD), strengthening U2AF65 and NTC recruitment to the pre-mRNA (David et al., 2011). Thus U2AF65/NTC tether the spliceosome and pre-mRNA to the CTD of elongating Pol II. By this mechanism the CTD enhances splicing, and describes interactions important for splicing and its coupling to transcription (David et al., 2011). In addition, such mechanism links Syf1 to transcription elongation, mRNA export, and to the nuclear excision DNA repair machinery in a transcription-coupled manner.

The human-to-yeast conserved TREX (TRanscription /EXport) complex shuttles mRNA from the nucleus to the cytoplasm. TREX is formed by the THO complex along with mRNA export proteins UAP56/Sub2 and Aly/Yra1. Strong evidence links TREX to transcription elongation in yeast. Strains carrying mutations in any of the four THO members fail to export mRNA and are defective in transcription elongation accumulating transcripts in or near their transcription start sites (Carmody and Wente, 2009). Furthermore, it has been shown that Syf1 acts as a novel transcription elongation factor required for TREX occupancy at transcribed genes (Chanarat et al., 2011). In mammals, TREX binds the mRNA co-transcriptionally suggesting that TREX is associated with the late stages of splicing. Whereas HPR1, a member of the THO complex, directly interacts with UAP56/Sub2 and is essential for UAP56/Sub2 and Aly/Yra1 recruiting to the mRNA (Zenklusen et al., 2002), in humans all THO components co-purify with spliceosomes (Jurica and Moore, 2003; Reed and Cheng, 2005).

Additionally, damage/alterations in DNA structure can interfere with DNA and RNA polymerases or compromise replication and transcription fidelity. Damaged DNA is restored by nuclear excision repair (NER). Two major NER pathways exist: global genome repair (GGR) and transcription-coupled repair (TCR). In the former, damage recognition is carried out by a DDB heterodimer which binds to the XPC-RAD23B-CEN2 complex (Hamann et al., 1995). In the later, proteins CSA and CSB together with Syf1 recognize DNA damage and recruit the DNA repairing machinery (Venema et al., 1990; Nakatsu et al., 2000). Both pathways converge in the following steps of DNA damage repair (Hanawalt and Spivak, 2008).

The protozoan parasite *Entamoeba histolytica* is the causative agent of amebiasis. *E. histolytica* infects approximately 1% of the human population, resulting in approximately 100,000 deaths annually (Nakada-Tsukui and Nozaki, 2016). Because pre-mRNA splicing mechanisms are almost unknown in this parasite, despite the limited amount of data, significant advances have been made to identify the Entamoeba splicing machinery and particular machineries (Miranda et al., 1996; Hernández-Rivas et al., 2000; Davis et al., 2007; Dávila López et al., 2008; Marchat et al., 2008; Hon et al., 2013; Valdés et al., 2014). Previous work using molecular biology and bioinformatic approaches revealed: splicing factor Prp6 (Hernández-Rivas et al., 2000), U2, U4, U5, and U6 snRNAs (Miranda et al., 1996; Davis et al., 2007), the possible lack of U1 snRNA in *E. histolytica* (Dávila López et al., 2008), the DExH/D RNA helicases involved in the proofreading of the sequential steps of spliceosome assembly and catalysis (Marchat et al., 2008), the preferred route of alternative splicing and the wide variety of alternative splice sites utilized by the Entamoeba spliceosome (Hon et al., 2013). Other proteins involved in pre-mRNA processing and maturation such as cleavage and specificity factors (CPSF160, 100, 73, and 30), cleavage stimulating factors (CstF77, 64, and 50), the cleavage factor Im of 25 kDa, both subunits of CFIIm (C1P1 and PCF11), FIP1, poly(A) polymerase, poly(A) binding protein, RBBP6 (Mpe1 in yeast), WDR33, PNAS-120, and PC4 (López-Camarillo et al., 2005, 2014). We have cloned HA-tagged snRNP component U1A and immunoprecipitated *in vivo* assembled pre-mRNA splicing complexes (Valdés et al., 2014). Among the nearly forty splicing factors identified by mass spectrometry were members of every splicing event, including factors involved in the formation of the aforementioned complexes A, B, and B^ACT^, as well as catalytic, post-catalytic, intron lariat and disassembly complexes (reviewed by Valdés et al., this issue). Of note is the E. histolytica putative ortholog of U2AF65, which participates in spliceosome/pre-mRNA (at the 3′ss of the intron)/CTD of Pol II. Factors of the *Entamoeba histolytica* NTC, Prp19 (EHI_13870), Cwc2 (EHI_126150), Cef1 (EHI_000550) and Syf (EHI_073300) were also identified. Furthermore, microarray data showed that EhSyf transcripts were overexpressed in amoebas transformed with HA-U1A (not shown) and splicing factor 1 (SF1). Trying to understand EhSyf participation in fundamental biological processes and to provide evidence about a key molecule that links transcription and splicing in a deep branched eukaryote, we cloned and characterized the general impact of EhSyf on splicing irrespective of 3′ss strength of Entamoeba virulence-related and virulence-unrelated introns.

## Materials and methods

### Entamoeba cultures

Axenic cultures of *E. histolytica* trophozoites strain HM-1:IMSS Cl-6 were incubated at 37°C in 13 × 100 mm screw-capped Pyrex glass tubes or plastic culture flasks in BI-S-33 medium as described (Diamond et al., 1972, 1978, 1995).

### Plasmid constructs and amoeba transfectants

The *EhSyf* gene was amplified by PCR using oligonucleotides containing appropriate restriction sites. PCR products were cloned in plasmids pEhExHA (Saito-Nakano et al., 2004) and pKT3M 04-trigger (Morf et al., 2013). To facilitate EhSyf cloning, a Sma I site was inserted in the pKT3M 04-trigger plasmid by site-directed mutagenesis. Constructs were verified by sequencing. Trophozoites were transformed with plasmids by liposome-mediated transfection as previously described (Nozaki et al., 1999). Transformants were selected with 5 or 10 μg/ml of Geneticin.

### Western blot and immunofluorescence

Protein extracts (60 μg) of amoeba transformants were separated by 10% SDS-PAGE and analyzed by western blotting using mouse anti-αSyf (human XAB2; Thermo Scientific), goat anti-Pol II (Santa Cruz Biotechnology), mouse anti-Actin, and secondary horseradish peroxidase-conjugated anti-IgG antibody (Sigma-Aldrich). The human anti-αSyf antibody target in EhSyf was validated by CLUSTALW alignment (Figure S1A). Enhanced chemiluminescent reagent (Perkin Elmer) was used to detect the proteins. To immunolocalize HA-EhSyf in amoeba transformants, cells were fixed and permeabilized in methanol at −20°C using anti-HA Alexa Fluor Labeled for viewing in a Zeiss LSM700 confocal microscope. All experiments were conducted in experimental and biological triplicates.

### RT-PCR

For overexpression, silencing and *in vivo* splicing assays EhSyf, Sam50, RabX13, MybS6, ClcB1, Cdc2, actin, and RNA polymerase II expression was monitored by RT-PCR. Total RNA of amoeba transformants was extracted with Trizol (Invitrogen) and the synthesis of cDNA was performed using the SuperScript III First Strand Synthesis System (Invitrogen) according to the manufacturer's instructions. Unspliced pre-mRNA/intron retained (IR) and mRNA molecules were detected with their respective primers (Table S1). Three biological replicates were done for each experiment. PCR products were resolved by electrophoresis and images of the gels were acquired in color inversion mode.

### Statistical and bioinformatics analyses

Phylogenetic trees were constructed with MEGA 7.0 (http://www.megasoftware.net/) using the neighbor-joining method; the significance of nodes was assessed using a bootstrap test with 1,000 (Tamura et al., 2013). To identify the type of TPR repeat consensus of EhSyf, CLUSTALW and CTREE alignments of the C-terminus of EhSyf (EHI_073300), *Homo sapiens XAB2* (accession number NP_064581), *Saccharomyces cerevisiae* (NP_010704), *Drosophila melanogaster* ORF de CG6197 accession number NP_610891, *Caenorhabditis elegans* ORF C50F2.3, accession number NP_491250, *Schizosaccharomyces pombe* ORF SPBC211.02c, accession number NP_596612 were carried out. EhSyf modeling was carried out with I-Tasser. For RT-PCR and western blots, differences between means were determined by *t*-test with GraphPad Prism 5.0. *p*-values were calculated comparing all pairs of the expression percentages obtained from the normalized IntDen values of densitometries using ImageJ. To eliminate plasmid transfection-effect, differences of transcript-expression values were normalized with respect to 18S rRNA (EhSyf) or RNA polymerase II (monitor transcripts) and were expressed as percent of the total expression (unspliced plus spliced gene products) as previously reported (Goren et al., 2006; de la Mata et al., 2010). Comparisons were carried out between empty vector and EhSyf constructs, prior verification of comparable copy number of plasmids by RT-PCR.

## Results

**Figure 1:**
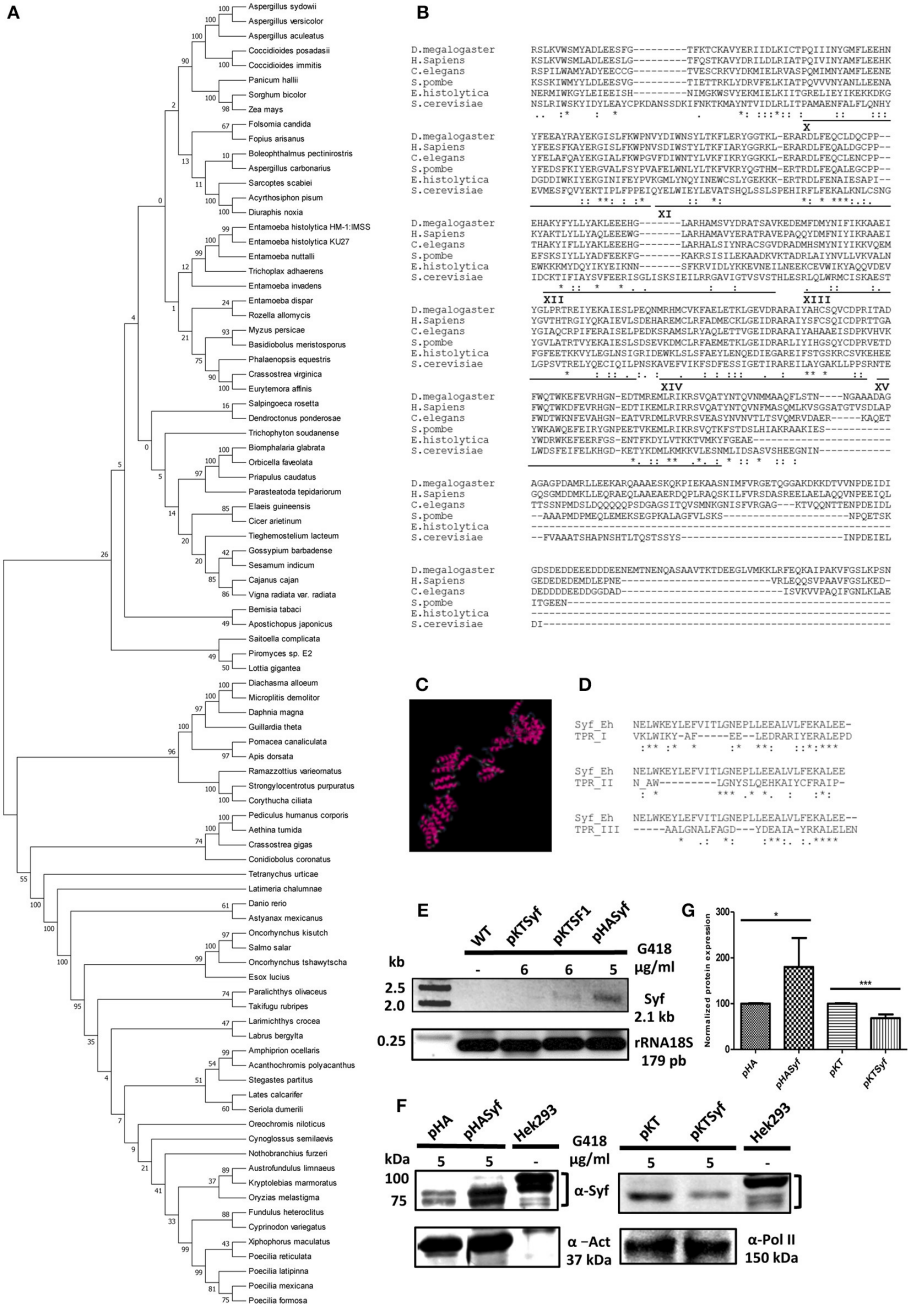
*In silico* and expression analysis of the *EhSyf* gene in amoeba transformants**. (A)** Evolutionary relationships of *EhSyf*. 89 protein sequences retrieved from EhSyf protein BLAST were used to construct the phylogenetic tree using MEGA 5.05 by the neighbor-joining method with 1,000 bootstrap replicates. **(B)** CLUSTALW alignments of the last 6 TPR motifs of EhSyf with Syf proteins from *Drosophila melanogaster, Homo sapiens, Caenorhabditis elegans, Schizosaccharomyces pombe* and *Saccharomyces cerevisiae*. **(C)** I-Tasser structure model of the 699 amino acids of EhSyf. **(D)** CTREE alignments of human TPR Types I-III motifs consensus sequences with EhSyf TPRs, showing that EhSyf possess Type I TPRs (Sikorski et al., 1991). **(E)** EhSyf silenced (pKTSyf) and over expressed (pHASyf) amoeba transformants were established by selection with the indicated amounts of G418 and Syf transcripts were monitored by RT-PCR. Basal post-transfection expression was compared to the Splicing Factor 1 silenced (pKTSF1) transfectants, and 18S rRNA was used for normalization. **(F)** Western blots were carried out with anti-αSyf (human) antibodies to assess protein overexpression and silencing of EhSyf in amoeba transformants, compared to the human Hek293 cell extracts. For normalization, actin and RNA polymerase II proteins were used. **(G)** Plot of normalized EhSyf protein overexpression (80%) and silencing (32%) in amoeba transformants. Error bars indicate the SD of three independent experiments, asterisks show statistically significant differences (t-Student *P* * < 0.05 *** < 0.001) compared to empty vectors.

We first performed phylogenetic inference of EhSyf. All EhSyf hits in the BLAST analysis were used to construct the rooted phylogeny tree (Figure 1A). As expected for E. histolytica proteins, the identity values of its relatives ranged from 20 to 30% (Table S2). The node of EhSyf relatives include the angiosperm *Phalaenopsis equestris*, the aphid *Myzus persicae*, the calanoid copepod *Eurytemora affinis*, the animal pathogen *Basidiobolus meristosporus, E. nuttalli, E. invadens, E. dispar*, other *E. histolytica* strains. Most importantly such node includes a representative of the deepest branching clade of fungi *Rozella allomycis*, and the only extant representative of basal metazoans *Trichoplax adhaerens* suggesting an early origin of EhSyf. This node appears somewhat distant from the rest of EhSyf relatives, however no other tetratricopeptide repeats (TPR) proteins were identified in the initial BLAST.

TPR proteins participate in a wide variety of functions involving protein-protein interactions (Das et al., 1998; Blatch and Lässle, 1999; Makiuchi et al., 2013). As deduced from the last six TPR motifs alignment with human, yeasts, insects and worms (Figure 1B), and *in silico* structural models, EhSyf contains 15 TPR class I (Sikorski et al., 1991) motifs in antiparallel α-helix arrays (Figures 1C,D). Interestingly, the C-terminus domain of EhSyf keeps low identity (I) but significant similarity (S) percentages with human (I 16.6%; S 36.4%), *S. cerevisiae* (I 19.3%; S 36.8%), *S. pombe* (I 25.2%; S 42.8%), *D. melanogaster* (I 16.9%; S 33.7%), and *C. elegans* (I 17.6%; S 35.2%).

**Figure 2:**
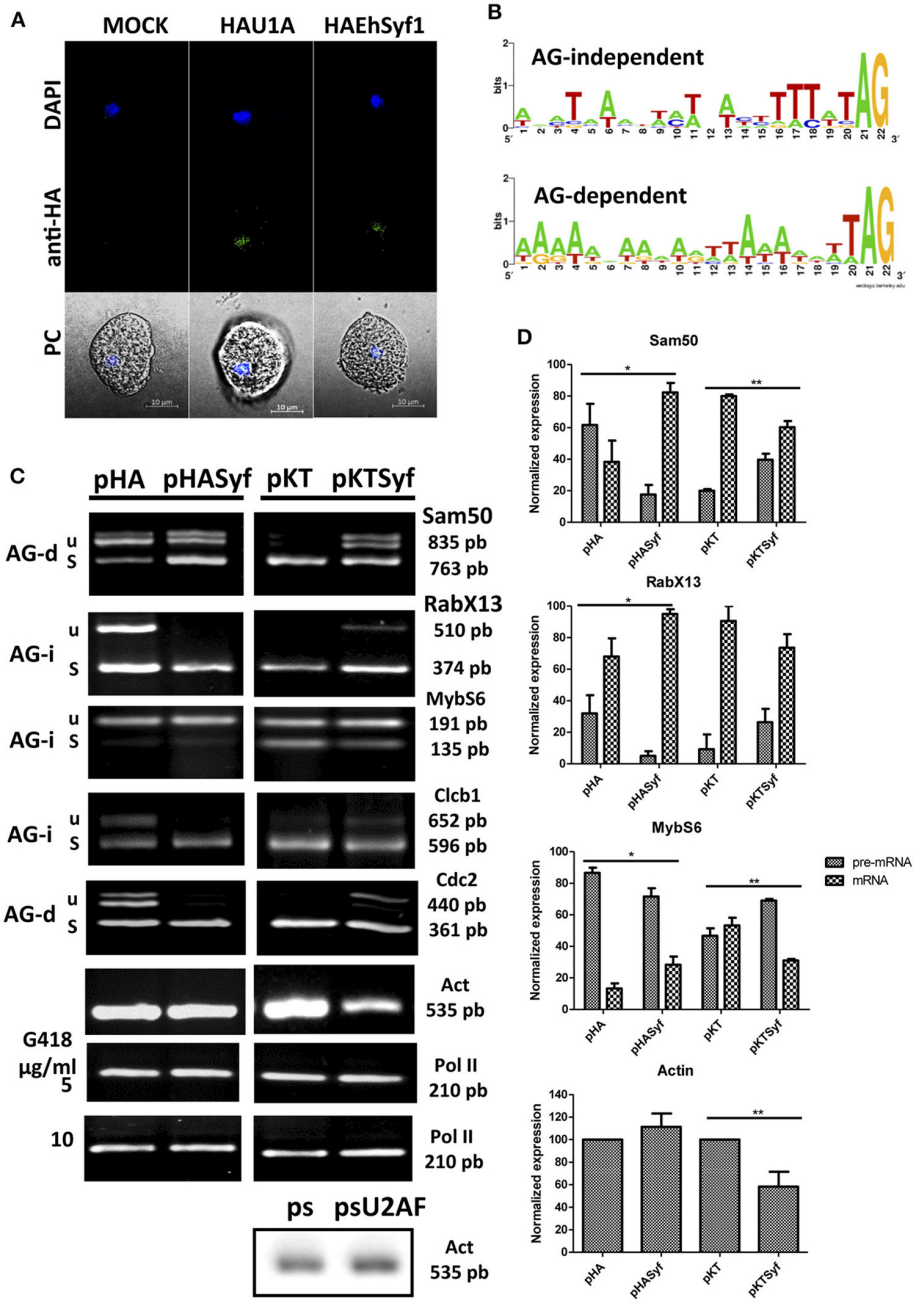
pre-mRNA splicing is favored in amoebae overexpressing EhSyf and defective with the knockdown**. (A)** *Entamoeba histolytica* HA-Syf transformants were plated onto microscopy slides, incubated with FITC-coupled anti-HA antibodies to detect appropriate nuclear localization of *EhSyf* protein by confocal microscopy. Nuclei were stained with DAPI. **(B)** WebLogos of the 3′ss of *E. histolytica* AG-independent (AG-i) and AG-dependent (AG-d) introns (Wu et al., 1999). **(C)** Representative semi-quantitative RT-PCR assays with specific primers to amplify Sam50, RabX13, MybS6, Clcb1, Cdc2 and the intron-less Actin gene products in 5 or 10 μg/ml G418 selected amoeba transformants. RNA polymerase II transcript was used to normalize the expression levels. For comparison, Actin gene expression was also monitored in U2AF splicing silenced amoebae (psU2AF). **(D)** Error bars indicate the SD of three independent experiments. The asterisks show significant differences (t-Student *P* * < 0.05 ** < 0.01) compared to empty vector transfectants. Whereas for Sam50, RabX13 and Actin the results shown correspond to HASyf expresser and knockdown transformants selected with 5 and 10 μg/ml G418, respectively; MybS6 gene selection of HASyf expression and knockdown figures show the data of transformants selected with 10 and 5 μg/ml G418, respectively. Clcb1 and Cdc2 gene products showed no significant differences in any selection (shown 5 μg/ml G418).

To partially understand EhSyf functions, pKT3M plasmid-based silencing (Morf et al., 2013) and HA-tagged over expression plasmid pEhExHA (Saito-Nakano et al., 2004) Syf Entamoeba transformants were established by selection with different amounts of geneticin, and their respective transcription and protein expression were monitored by RT-PCR and western blot. Contrary to transcriptomic analyses reported in AmoebaDB, we were not able to detect wild type EhSyf mRNA (Figure 1E), suggesting that Entamoeba expresses very low amounts of EhSyf. This is consistent with our previous findings in which EhSyf was not detected in MS2 aptamer-tagged RabX13 intron immunoprecipitates, which contained components of splicing B and C complexes, although other members of U2 snRNP and NTC were detected (Valdés et al., 2014; Valdes et al. this issue), reflecting NTC rearrangements within the spliceosome during pre-spliceosome to pre-catalytic complex transition. In spite of our observations, and because we expect constitutive EhSyf expression, we ensured the lack of EhSyf by means of pKTSyf-mediated silencing. To eliminate off-target effect another splicing-related protein SF1 (pKTSF1) was silenced, and we observed that EhSyf mRNA expression was enhanced, instead of reduced. Conversely, more EhSyf mRNA was detected in pHASyf overexpression transformants. In agreement with this, whereas the ≈ 84 kDa EhSyf was robustly expressed in mock-transfected amoebae (pHA), pHASyf transformants expressed 80% more, nearly as abundant as that of Hek293 human cells (Figure 1F, Figures S1A–D). Although we cannot ascertain EhSyf mRNA reduction, only 32% reduction of EhSyf protein expression was achieved in pKTSyf amoeba transformants (Figure 1G). Finally, anti-HA confocal microscopy immunofluorescence experiments showed that EhSyf localized to the nucleus similar to the HA-U1A splicing factor control (Figure 2A and Figure S1E).

To determine its role in splicing, we reasoned that slight changes of EhSyf expression, being part of the NTC, would imbalance wild type pre-mRNA/mRNA ratios in a general manner. Despite the capacity of U1 snRNP components to define the 5′ss and of the U2AF dimer to define the 3′ss. These protein/RNA interactions must be taken into consideration since most 5′ss (GUUUGUUU) are strong and conserved in Entamoeba (Hon et al., 2013), in the absence of U1 snRNA RNA (Dávila López et al., 2008). Therefore 5′ss definition must be carried out by U1 snRNP components and splicing auxiliary factors as described for other systems (Förch et al., 2002; Huang et al., 2012). For 3'ss definition, whereas strong (AG-independent) poly-pyrimidine tracts do not always require U2AF35, both U2AF35/65 are required to define AG-dependent 3'ss with weak poly-pyrimidine tracts (Wu et al., 1999). A collection of introns was analyzed with respect to their AG-dependency (Figure 2B) and several were chosen to test our hypothesis. AG-dependent introns of *Sam50* and *Cdc2*, and AG-independent introns of *RabX13, MybS6* and intron 1 of *ClcB*. As a control the intron-less Actin transcript was analyzed. These introns were also selected because they represent both virulence-related [*Sam50, MybS6*, and *ClcB* are downregulated after trophozoites recuperated from hamster liver abscesses (Weber et al., 2016)] and virulence-unrelated transcripts. Furthermore, in steady state conditions, they always produce unspliced pre-mRNA/intron retained (IR) and mRNA variants facilitating the monitoring of splicing variats. *In vivo* splicing assays were carried out using RNA extracts from the EhSyf expresser and silenced transformants. Compared to the HA control, spliced mRNA increased in all HASyf expresser transformants at the expense of pre-mRNA/IR variants. Correspondingly, EHSyf silencing resulted in pre-mRNA/IR accumulation at the expense of mRNA, compared to the pKT silencing controls (Figure 2C). Statistically different transcription levels are shown (Figure 2D). Unexpectedly this approach allowed us to detect that high selective pressure EhSyf silencing affected Actin gene expression too. This change was not observed in U2AF-silenced ameba transformants (psU2AF) selected with the same amount of G418 (Figures 2B,C), indicating that low amounts of EhSyf impact transcription of intron-less genes too.

## Discussion

*EhSyf* gene encodes a tetratricopeptide repeats protein involved in several nuclear functions. The Entamoeba *Syf* gene products appear to have low copy number that can be boosted during transfection of unrelated genes. This might reflect the direct/indirect participation of EhSyf in transcription, or it might reflect that the introduction of plasmid DNA evokes general transcription/DNA-repair mechanisms, increasing in turn *EhSyf* transcripts. The impact of plasmid transfection on endogenous genes has been documented since the pioneering works attempting heterologous expression of proteins in *E. histolytica* (Hamann et al., 1995).

EhSyf expression directly impacts splicing in a general manner. This reflects its relationship with the large subunit of U2AF which binds to poly-pyrimidine tracts in front of the 3′ss, and tethers the pre-mRNA/spliceosome to the CTD. Interestingly, EhSyf silencing but not overexpression, negatively affected transcription even of intron-less genes indicating its role in transcription elongation.

Together, our data suggest the coordinated functions of EhSyf in transcription and splicing in this early branched protist. Although the impact of EhSyf in transcription termination and mRNA export still remains to be tested. We conclude that EhSyf is a *bona fide Entamoeba histolytica* Syf1 ortholog that participates in splicing and transcription elongation. To our knowledge this is the first evidence of the molecular link between transcription and splicing in *Entamoeba histolytica*.

## Author contributions

JV, DT-C conception, experimental design, manuscript preparation; DT-C, JG-R, OS-C, and JV data acquisition and interpretation.

### Conflict of interest statement

The authors declare that the research was conducted in the absence of any commercial or financial relationships that could be construed as a potential conflict of interest.
