# Supplementary material for: The Entamoeba histolytica Syf1 Homolog Is Involved in the Splicing of AG-Dependent and AG-Independent Transcripts
Source: Front Cell Infect Microbiol. 2018 Jul 9;8:229. doi: 10.3389/fcimb.2018.00229 (PMC6046404; doi:10.3389/fcimb.2018.00229)
Supplement: Supplementary file 1 [file Table_1.pdf]

Supplemental data

Table S1. Oligonucleotides used in this work.

Table S2. PSI-BLAST hits using EhSyf (EHI_073300) amino acid sequence as query.

Figure S1. (A and B) Western blot performed with anti-αSyf (XAB2 *Homo Sapiens*), anti-actin and anti-RNA polymerase II antibodies on protein extracts of amoeba transformants and Hek293 cell extracts as positive control. The arrows and brackets indicate the relevant proteins and their post translationally modified forms. **(C)** Western blot of the human Hek293 and HELA cell extracts were carried out anti-αSyf antibodies. Multiple TPR motifs are detected by the Syf1 antibody in HeLa cells. **(D)** CLUSTALW alignment of anti-αSyf1 target sequence (amino acids 30 – 243) with EhSyf. **(E)** Lower magnification of the confocal microscopy images.

Table S1

Gene id	Oligo	Sequce (5´→ 3´)
rRNA 18S	FRibEh239F	ATTGTCGTGGCATCCTAACTCA
	RRibEh88R	GCGGACGGCTCATTATAACA
Actin EHI_107290	EhACT F	GAGCTGTATTCCCATCCATTGTTG
	EhACT R	CTTTCAGCAGTAGTGGTGAAAGC
Cdc2 EHI_065280	Cdc2 F	CAATTAGGAGAAGGAACATATGG
	Cdc2 R	GTGGTTTCATATCTCTGTGAAG
Clc-B EHI_186860	Chlo F	ATGGAACAAAATTTACCCTC
	ChlRE2	CACCATAATCCACTTCCAATAG
RabX13 EHI_065790	Rab2 F	CGTTGTTGGAGACTCTTCAGTTGG
	Rab2R	GACCCATTTCAGTTGAAACAGTTC
Sam50 EHI_078220	FSam50	GCAATGACAACAAGAATGCAA
	RSam50	AAGAAACCCCAACTCCACAA
EhMybS6 EHI_155580	S6 FWD	TCAAGTTCGTTCTCATGCAC
	S6 REV	CTAGAACAATTGGTTCGAAGG
POL II EHI_056690	PolIIF	GATCCAACATATCCTAAAACAACA
	PolIIR	TCAATTATTTTCTGACCCGTCTTC
EhSyf EHI_073300	SyfSma	TCCCCCGGGATGAATGAAGAAAAGAATATTTC
	SyfXho	CAACTCGAGTCATTCTGCTTCTCCAAAAT
	Syf2F	GAAAGTCTTACTGTCCAAGA
	Syf2R	TGAATTGTTTTAATTCCTTC

	Description	Ident	Accession
1	Hypothetical protein, conserved [Entamoeba histolytica HM-1:IMSS]	100%	XP_656694.1
2	Hypothetical protein EHI5A_009350 [Entamoeba histolytica KU27]	99%	EMD44721.1
3	Hypothetical protein ENU1_204310 [Entamoeba nuttalli P19]	98%	XP_008860429.1
4	Hypothetical protein [Entamoeba dispar SAW760]	94%	XP_001736363.1
5	pre-mRNA-splicing factor SYF1, putative [Entamoeba invadens IP1]	45%	XP_004183662.1
6	PREDICTED: pre-mRNA-splicing factor SYF1 [Acyrthosiphon pisum]	30%	XP_001951071.2
7	PREDICTED: pre-mRNA-splicing factor SYF1 [Diuraphis noxia]	30%	XP_015376632.1
8	Hypothetical protein PIROE2DRAFT_44827 [Piromyces sp. E2]	30%	OUM61915.1
9	pre-mRNA-splicing factor SYF1-like protein [Sarcoptes scabiei]	30%	KPM02154.1
10	PREDICTED: pre-mRNA-splicing factor SYF1 [Bemisia tabaci]	30%	XP_018907859.1
11	putative pre-mRNA-splicing factor SYF1 isoform X1 [Apostichopus japonicus]	30%	PIK47966.1
12	Hypothetical protein LOTGIDRAFT_236450 [Lottia gigantea]	29%	XP_009065584.1
13	pre-mRNA-splicing factor SYF1 [Myzus persicae]	29%	XP_022174492.1
14	Hypothetical protein GOBAR_AA31617 [Gossypium barbadense]	29%	PPR89062.1
15	PREDICTED: pre-mRNA-splicing factor SYF1 [Austrofundulus limnaeus]	29%	XP_013880197.1
16	Hypothetical protein C0Q70_17885 [Pomacea canaliculata]	29%	PVD22082.1
17	PREDICTED: pre-mRNA-splicing factor SYF1-like [Biomphalaria glabrata]	29%	XP_013080345.1
18	TPR-like helical domain-containing protein [Tieghemostelium lacteum]	29%	KYQ93672.1
19	Hypothetical protein ASPSYDRAFT_145789 [Aspergillus sydowii CBS 593.65]	29%	OJJ61987.1
20	PREDICTED: pre-mRNA-splicing factor SYF1 [Microplitis demolitor]	29%	XP_008547181.1
21	pre-mRNA-splicing factor SYF1 [Parasteatoda tepidariorum]	29%	XP_015925910.1
22	pre-mRNA-splicing factor SYF1 isoform X2 [Boleophthalmus pectinirostris]	29%	XP_020791053.1
23	PREDICTED: pre-mRNA-splicing factor SYF1-like [Tetranychus urticae]	29%	XP_015791103.1
24	PREDICTED: pre-mRNA-splicing factor SYF1 [Nothobranchius furzeri]	29%	XP_015803968.1
25	pre-mRNA-splicing factor SYF1 [Xiphophorus maculatus]	29%	XP_005815543.1
26	PREDICTED: pre-mRNA-splicing factor SYF1 [Poecilia reticulata]	29%	XP_008414255.1
27	PREDICTED: pre-mRNA-splicing factor SYF1 [Poecilia mexicana]	29%	XP_014838858.1
28	PREDICTED: pre-mRNA-splicing factor SYF1 [Esox lucius]	29%	XP_010890822.1
29	PREDICTED: pre-mRNA-splicing factor SYF1 [Poecilia formosa]	29%	XP_007560418.1
30	PREDICTED: pre-mRNA-splicing factor SYF1 [Larimichthys crocea]	29%	XP_010728663.2
31	PREDICTED: pre-mRNA-splicing factor SYF1 [Poecilia latipinna]	29%	XP_014875910.1
32	pre-mRNA splicing factor Syf-1 [Basidiobolus meristosporus CBS 931.73]	29%	ORX91893.1
33	Hypothetical protein ASPVEDRAFT_77853 [Aspergillus versicolor CBS 583.65]	29%	OJI96079.1
34	Hypothetical protein ASPCADRAFT_206302 [Aspergillus carbonarius ITEM 5010]	29%	OOF97470.1
35	pre-mRNA-splicing factor SYF1 [Labrus bergylta]	29%	XP_020496596.1
36	pre-mRNA-splicing factor SYF1 isoform X1 [Amphiprion ocellaris]	29%	XP_023144374.1
37	XPA-binding protein 2, putative [Coccidioides posadasii C735 delta SOWgp]	29%	XP_003067160.1
38	pre-mRNA-splicing factor SYF1 [Oryzias melastigma]	29%	XP_024128676.1
39	XPA-binding protein, putative [Pediculus humanus corporis]	29%	XP_002432456.1
40	pre-mRNA-splicing factor SYF1 [Danio rerio]	29%	NP_001038248.1
41	PREDICTED: pre-mRNA-splicing factor SYF1 [Kryptolebias marmoratus]	29%	XP_017284774.1
42	pre-mRNA-splicing factor syf1 [Coccidioides immitis RS]	29%	XP_001239579.2
43	Pre-mRNA-splicing factor SYF1 [Crassostrea gigas]	29%	EKC32877.1
44	Hypothetical protein ASPACDRAFT_1902702 [Aspergillus aculeatus ATCC 16872]	29%	XP_020055131.1
45	PREDICTED: pre-mRNA-splicing factor SYF1 [Lates calcarifer]	29%	XP_018560087.1
46	PREDICTED: pre-mRNA-splicing factor SYF1 [Takifugu rubripes]	29%	XP_003964804.1
47	pre-mRNA-splicing factor SYF1-like [Crassostrea virginica]	29%	XP_022325906.1
48	Pre-mRNA-splicing factor SYF1 [Salmo salar]	29%	NP_001167289.1
49	pre-mRNA-splicing factor SYF1-like [Eurytemora affinis]	29%	XP_023325255.1
50	PREDICTED: pre-mRNA-splicing factor SYF1 isoform X1 [Strongylocentrotus purpuratus]	29%	XP_003727542.1
51	pre-mRNA-splicing factor SYF1-like [Oncorhynchus tshawytscha]	29%	XP_024259840.1
52	pre-mRNA-splicing factor SYF1 [Seriola dumerili]	29%	XP_022608380.1
53	PREDICTED: pre-mRNA-splicing factor SYF1 [Dendroctonus ponderosae]	29%	XP_019757315.1
54	PREDICTED: pre-mRNA-splicing factor SYF1-like [Apis dorsata]	29%	XP_006608420.1
55	pre-mRNA-splicing factor syf1 [Trichophyton soudanense CBS 452.61]	29%	EZF75879.1
56	SYF1 [Corythucha ciliata]	29%	ARW29622.1
57	protein Cwf3 [Schizosaccharomyces pombe]	29%	NP_596612.1
58	Hypothetical protein GUITHDRAFT_75030 [Guillardia theta CCMP2712]	28%	XP_005828114.1
59	Hypothetical protein G7K_1202-t1 [Saitoella complicata NRRL Y-17804]	28%	GAO46988.1
60	pre-mRNA-splicing factor SYF1 [Sorghum bicolor]	28%	XP_002463280.1
61	PREDICTED: LOW QUALITY PROTEIN: pre-mRNA-splicing factor SYF1 [Elaeis guineensis]	28%	XP_010937810.1
62	pre-mRNA-splicing factor SYF1 [Phalaenopsis equestris]	28%	XP_020574450.1
63	PREDICTED: pre-mRNA-splicing factor SYF1 [Cicer arietinum]	28%	XP_004485713.1
64	uncharacterized protein LOC100217127 [Zea mays]	28%	NP_001337639.1
65	Hypothetical protein TRIADDRAFT_21689 [Trichoplax adhaerens]	28%	XP_002109964.1
66	PREDICTED: pre-mRNA-splicing factor SYF1 [Diachasma alloeum]	28%	XP_015122343.1
67	Hypothetical protein PAHAL_C04329 [Panicum hallii]	28%	PAN15143.1
68	Pre-mRNA-splicing factor SYF1 [Cajanus cajan]	28%	KYP63008.1
69	PREDICTED: pre-mRNA-splicing factor SYF1 isoform X1 [Paralichthys olivaceus]	28%	XP_019949919.1
70	pre-mRNA-splicing factor SYF1-like [Fundulus heteroclitus]	28%	XP_021180250.1
71	pre-mRNA-splicing factor SYF1-like [Folsomia candida]	28%	XP_021947866.1
72	pre-mRNA-splicing factor SYF1 [Sesamum indicum]	28%	XP_011093520.1
73	PREDICTED: pre-mRNA-splicing factor SYF1 [Fopius arisanus]	28%	XP_011309944.1
74	pre-mRNA-splicing factor SYF1-like [Orbicella faveolata]	28%	XP_020625116.1
75	pre-mRNA-splicing factor SYF1-like [Vigna radiata var. radiata]	28%	XP_014515558.1
76	PREDICTED: pre-mRNA-splicing factor SYF1 [Cyprinodon variegatus]	28%	XP_015227467.1
77	PREDICTED: pre-mRNA-splicing factor SYF1 [Cynoglossus semilaevis]	28%	XP_008327835.1
78	Anaphase-promoting complex subunit 11 domain-containing protein [Rozella allomycis CSF55]	28%	EPZ34667.1
79	PREDICTED: pre-mRNA-splicing factor SYF1 [Oreochromis niloticus]	28%	XP_003442140.1
80	pre-mRNA-splicing factor SYF1 [Acanthochromis polyacanthus]	28%	XP_022075386.1
81	pre-mRNA-splicing factor SYF1 [Astyanax mexicanus]	28%	XP_022523751.1
82	PREDICTED: pre-mRNA-splicing factor SYF1-like [Priapulus caudatus]	28%	XP_014663513.1
83	pre-mRNA-splicing factor SYF1-like [Oncorhynchus kisutch]	28%	XP_020348748.1
84	Pre-mRNA-splicing factor SYF1 [Daphnia magna]	28%	KZS18453.1
85	PREDICTED: pre-mRNA-splicing factor SYF1 [Stegastes partitus]	28%	XP_008297878.1
86	PREDICTED: pre-mRNA-splicing factor SYF1 isoform X1 [Latimeria chalumnae]	28%	XP_006008659.1
87	PREDICTED: pre-mRNA-splicing factor SYF1 [Aethina tumida]	28%	XP_019864746.1
88	Hypothetical protein RvY_07468 [Ramazzottius varieornatus]	28%	GAU95952.1
89	pre-mRNA-splicing factor SYF1 [Salpingoeca rosetta]	27%	XP_004991470.1
90	pre-mRNA-splicing factor SYF1-like protein [Conidiobolus coronatus NRRL 28638]	27%	KXN72905.1
91	fandango [Drosophila melanogaster]	27%	NP_610891.1
92	pre-mRNA-splicing factor SYF1 [Homo sapiens]	26%	NP_064581.2
93	SYF pre-mRNA splicing factor homolog [Caenorhabditis elegans]	26%	NP_491250.1
94	mRNA splicing protein SYF1 [Saccharomyces cerevisiae S288C]	20%	NP_010704.1

Table S2
