# Supplementary material for: The Entamoeba histolytica Syf1 Homolog Is Involved in the Splicing of AG-Dependent and AG-Independent Transcripts
Source: Front Cell Infect Microbiol. 2018 Jul 9;8:229. doi: 10.3389/fcimb.2018.00229 (PMC6046404; doi:10.3389/fcimb.2018.00229)
Supplement: Supplementary file 2 [file Image_1.pdf]

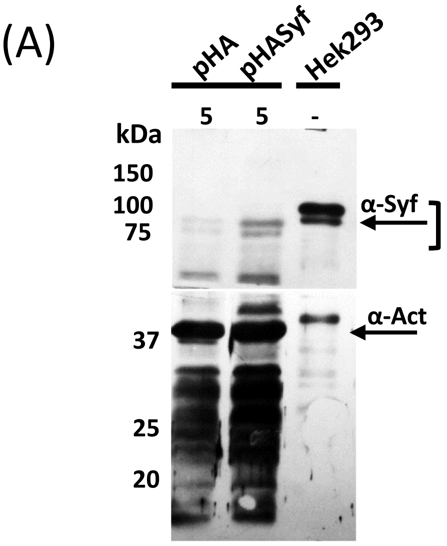

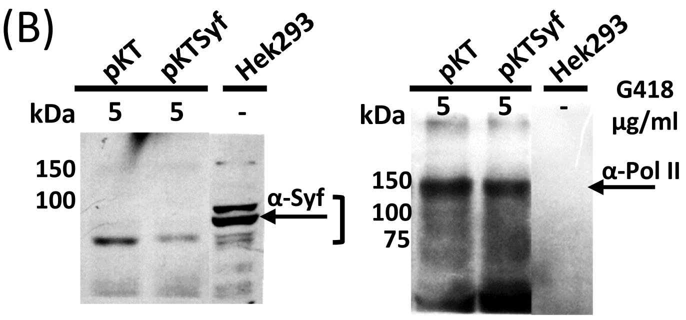

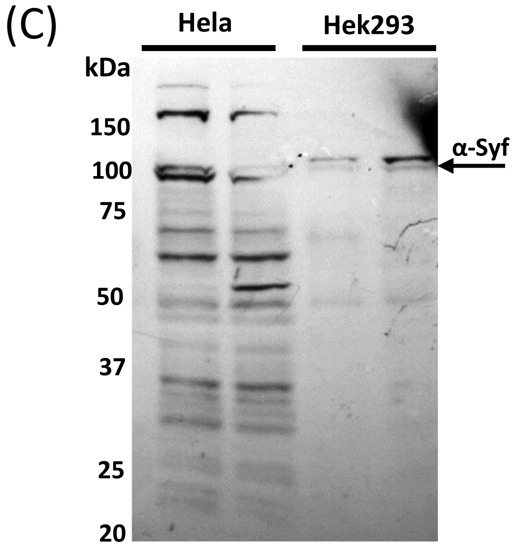

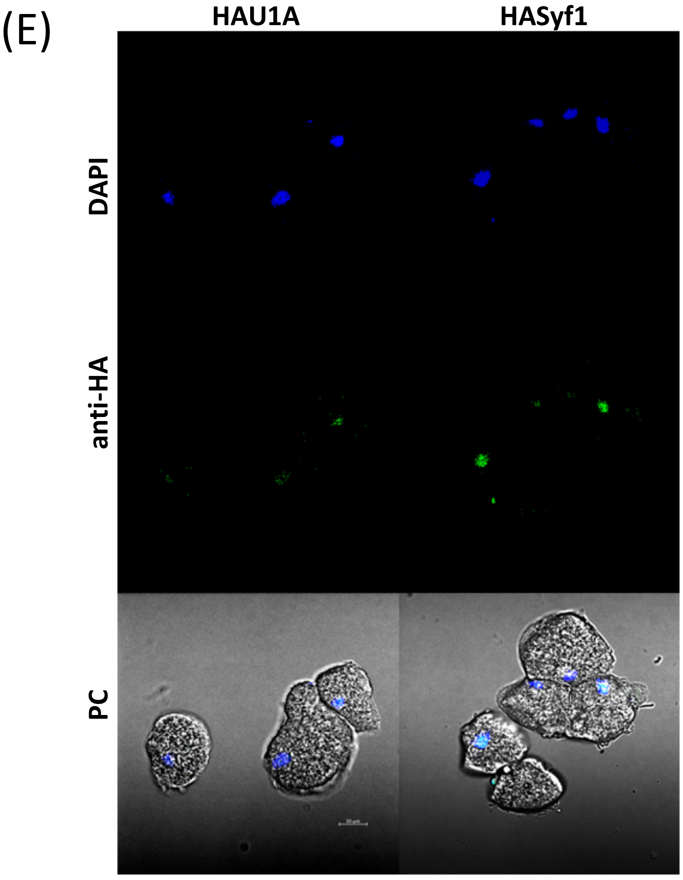

(D)

```
E.histolytica        MNEEKNISIYEFDVKQNPQSFKTWWNYIEYFDESHFQSKITIFQRALHELPGSYKLWYHY
α-Syf H.sapiens      ---------------RNQFSVKCWLRYIEFKQGAPKPRLNQLYERALKLLPCSYKLWYRY
                                    :*  *.* * .***: : :       :::***: ** ******:*

E.histolytica        LQTILINARKSGIDTEIRKSVNEVFEESLVYMNKMPVIWKLYIEWLIENGEITQMRRVFD
α-Syf H.sapiens      LKARRAQVKHRCVTDPAYEDVNNCHERAFVFMHKMPRLWLDYCQFLMDQGRVTHTRRTFD
                     *::   :.::  :     :.**: .*.::*:*:*** :*  * ::*:::*.:*: **.**

E.histolytica        RSLQSLPIGQHNELWKVVMKFVITLNTPLLFEKLVLRHILLDRGMIGEYIQICKKKGEKY
α-Syf H.Sapiens      RALRALPITQHSRIWPLYLRFLRSHPLPETAVRGYRRFLKLSPESAEEYIEYLKSSD---
                     *:*::*** **..:* : ::*: :   *    :   *.: *.     ***:  *...
```
